# Diagnostic value of D-dimer to fibrinogen ratio for pulmonary embolism in postpartum women

**DOI:** 10.1186/s12884-024-06670-1

**Published:** 2024-07-16

**Authors:** Wenting Zhou, Cuicui Qu, Xiaohua Liu, Junfeng Huang

**Affiliations:** 1 Department of Obstetrics and Gynecology, Shanghai Key Laboratory of Maternal Fetal Medicine, Shanghai Institute of Maternal-Fetal Medicine and Gynecologic Oncology, Shanghai First Maternity and Infant Hospital, School of Medicine, Tongji University, No.2699 West Gaoke Road, Pudong New Area, Shanghai, 200092 P.R. China; 2 Department of Intensive Care Medicine, Zhongshan Hospital, Fudan University, 180 Yi Xue Yuan Road, Shanghai, 200032 P.R. China

**Keywords:** Pulmonary embolism, Postpartum, D-dimer to fibrinogen ratio, Diagnostic value

## Abstract

**Background:**

Pulmonary embolism is a common disease associated with high mortality and morbidity. Diagnosing pulmonary embolism is challenging due to diverse clinical presentations and the lack of specific biomarkers. The study aimed to investigate the diagnostic value on pulmonary embolism for postpartum women by D-dimer to fibrinogen ratio, and it combined with neutrophil-to-lymphocyte ratio or platelet-to-lymphocyte ratio.

**Methods:**

A total of 537 women with suspected pulmonary embolism were selected as the research subjects from the Shanghai First Maternity and Infant Hospital between 1 January 2019 and 31 October 2022. The D-dimer to fibrinogen ratio and it combined with neutrophil-to-lymphocyte ratio or platelet-to-lymphocyte ratio were applied to evaluate the clinical probability of pulmonary embolism, and the positive predictive value of both scores were calculated using computed tomography pulmonary arteriography as a gold standard. The diagnostic value of D-dimer to fibrinogen ratio, combined with neutrophil-to-lymphocyte ratio or platelet-to-lymphocyte ratio was evaluated by the area under the curve, sensitivity, specificity, and other indicators in the receiver operator characteristic curve.

**Results:**

Among the 502 women included for analysis, 194 (38.65%) were definitely diagnosed as pulmonary embolism. The positive predictive values of D-dimer to fibrinogen ratio and it combined with platelet-to-lymphocyte ratio or neutrophil-to-lymphocyte ratio were 70.1%, 50.5%, and 56.5%, respectively in the postpartum women, the area under the curve for the D-dimer to fibrinogen ratio and it combined with platelet-to-lymphocyte ratio or neutrophil-to-lymphocyte ratio were 0.606 (95%CI: 0.562–0.650), 0.624 (95%CI: 0.575–0.673), and 0.639 (95%CI: 0.592–0.686), respectively. The negative predictive values of D-dimer to fibrinogen ratio, it combined with platelet-to-lymphocyte ratio or neutrophil-to-lymphocyte ratio were 50.5%, 70.1%, and 69.8%, respectively.

**Conclusion:**

The diagnostic value of the D-dimer to fibrinogen ratio was higher than the D-dimer for the postpartum women with suspected pulmonary embolism. The combination of either the neutrophil-to-lymphocyte ratio or the platelet-to-lymphocyte ratio with D-dimer t**o** fibrinogen ratio is an appropriate strategy to rule out pulmonary embolism.

## Background

Pulmonary embolism (PE) represents a life-threatening condition characterized by the obstruction of pulmonary arteries due to blood clots, typically originating from deep vein thrombosis [1, 2]. During pregnancy and the immediate postpartum period, women face an elevated risk of PE due to factors including hypercoagulability, stasis, and endothelial damage [3]. The risk of PE increases in later pregnancy, peaking in the immediate postpartum phase and persisting for 6–12 weeks postpartum, being one of the leading causes of maternal mortality [4, 5]. Early diagnosis and treatment of PE are paramount to prevent potentially fatal complications. Diagnosing PE in postpartum women proves challenging, as symptoms like dyspnea and chest pain can be nonspecific and overlap with other postpartum conditions [6]. Thus, there is a need for reliable biomarkers that can aid in the timely and accurate diagnosis of PE in postpartum women.

D-dimer and fibrinogen are two biomarkers involved in the coagulation pathway that show promise in diagnosing PE [7, 8]. D-dimer is a fibrin degradation product released when a blood clot dissolves, while fibrinogen is a key protein involved in blood clot formation. Some studies have found that D-dimer is useful for ruling out PE in pregnant and postpartum women [9–11]. However, D-dimer levels can be affected by many factors and may be elevated during pregnancy, potentially increasing false positive rates and leading to unnecessary imaging tests and anticoagulant therapy [12, 13]. D-dimer to fibrinogen ratio (DFR) is a valuable predictor of PE, and combining D-dimer with fibrinogen can enhance the specificity of D-dimer and improve accuracy [7, 14]. Studies have demonstrated the value of DFR in predicting PE in patients in the emergency department [15, 16]. Anyway, the diagnostic value of DFR in postpartum women suspected of having PE still unclear.

Our study aimed to investigate the diagnostic value of DFR in identifying PE in postpartum women. Previous studies have reported the predictive value of NLR or PLR on postpartum depression and poor neonatal prognosis [17–19]. Given that neutrophil-to-lymphocyte ratio (NLR) and platelet-to-lymphocyte ratio (PLR) are simple biomarkers readily available from routine laboratory values, and may be useful components of PE risk prediction models, we would further investigate the diagnostic value of DFR combined with NLR or PLR in identifying PE in postpartum women.

## Methods

### Study population

Postpartum women with suspected PE who visited Shanghai First Maternity and Infant Hospital, between 1 January 2019 and 31 October 2022, were enrolled in this cross-sectional study. The study protocol was approved by the Ethics Committee of Shanghai First Maternity and Infant Hospital (No. KS21252). The need for written informed consent was waived by the Ethics Committee of Shanghai First Maternity and Infant Hospital due to retrospective nature of the study. All methods were performed in accordance with the relevant guidelines and regulations.

The inclusion criteria were as follows: (1) age ≥ 18 years old, (2) postpartum women (in 6 weeks after delivery) with suspected PE, (3) having complete clinical data. Postpartum women were excluded of those (1) who did not receive computed tomography pulmonary angiography (CTPA) examinations (including patients who were allergic to intravenous enhanced contrast agents or have other contraindications to performing CTPA), (2) who did not have D-dimer and fibrinogen assessment, (3) treatment transferred to another hospital.

### Data collection

The case information was collected by obstetricians and nurses in our hospital. Prior to the study, all researchers received uniform training to ensure the quality of case collection. Sociodemographic data, laboratory tests, vital signs, and pregnancy-related data were obtained for further analysis. Sociodemographic data included age, height, weight, family disease history, previous disease history, complications, and body mass index (BMI). Laboratory tests included hemoglobin, red blood cell, white blood cell, platelet, neutrophil, lymphocyte, monocyte, eosinophile, basophilie, mean platelet volume, red blood cell distribution width-coefficient of variation, prothrombin time, activated partial thromboplastin time, thrombin time, NLR, PLR, and fibrinogen. Pregnancy-related data included parturition, abortion, gestation, premature delivery, delivery mode, number of fetuses, and anticoagulant therapy. Anticoagulant therapy included prophylactic therapy during pregnancy and postpartum routine anticoagulant therapy.

### D-dimer and fibrinogen measure

D-dimer were measured by a high sensitive turbidimetric immunoassay (STA-R analyzer). The fibrinogen concentration was determined using the same analyzer. Both the D-dimer and fibrinogen were detected in the same laboratory. Measure on D-dimer and fibrinogen were within 24 h after clinicians identified postpartum woman with suspected PE.

### PE assessment

CTPA was used for PE diagnosis. Signs of PE detected in the pulmonary artery included central eccentric partial filling defects encircled by contrast medium, complete vessel section occupancy by filling defects, and mural defects. A 64-row multidetector CT scanner (Lightspeed VCT, GE Healthcare) was utilized for the performance of CTPA.

### Sample size

The sample size was inevitably determined by the incidence of diagnosed and suspected PE during the data collection period. Based on previous studies, the area under curve (AUC) of D-dimer level in PE diagnosis in suspected PE patients was 0.735 [4]. We assumed that the expected AUC of DFR was ≥ 0.8, α was 0.05, detection power (1-β) was 0.8, and sample size was 496 cases calculated by PASS 11.0. Considering a shedding rate of 10%, a total sample size of 551 was needed in this study.

### Statistical analysis

The normality of quantitative data was tested using skewness and kurtosis, and the equality of variances were tested using Levene tests. Quantitative data. Continuous data with normal distribution were described as means and standard deviation, non-normal distribution were expressed as median and interquartile. Comparison between two groups were conducted using Student’s t tests, Satterthwaite t test, and Wilcoxon rank sum tests. Categorical data were expressed as numbers and percentage (%). Chi-square tests and Fisher exact tests were used for comparison of categorical data. Potential covariates were selected using weighted univariate logistic regression models and stepwise regression methods. The relationship between DFR levels and PE diagnosis were explored using weighted univariate and multivariate logistic regression models, and results were shown with odds ratios (ORs) and 95% confidence intervals (CIs). Correlation between PLR and NLR was detected using Pearson correlation. Diagnostic accuracy of DFR, DFR combined with NLR or PLR was assessed by plotting a receiver-operator characteristic (ROC) curve and calculating the AUC. R version 4.2.3 (2023-03-15 ucrt) were utilized for all statistical analysis and *P* < 0.05 was considered statistical significance.

## Results

### Characteristic of postpartum women

**Fig. 1:**
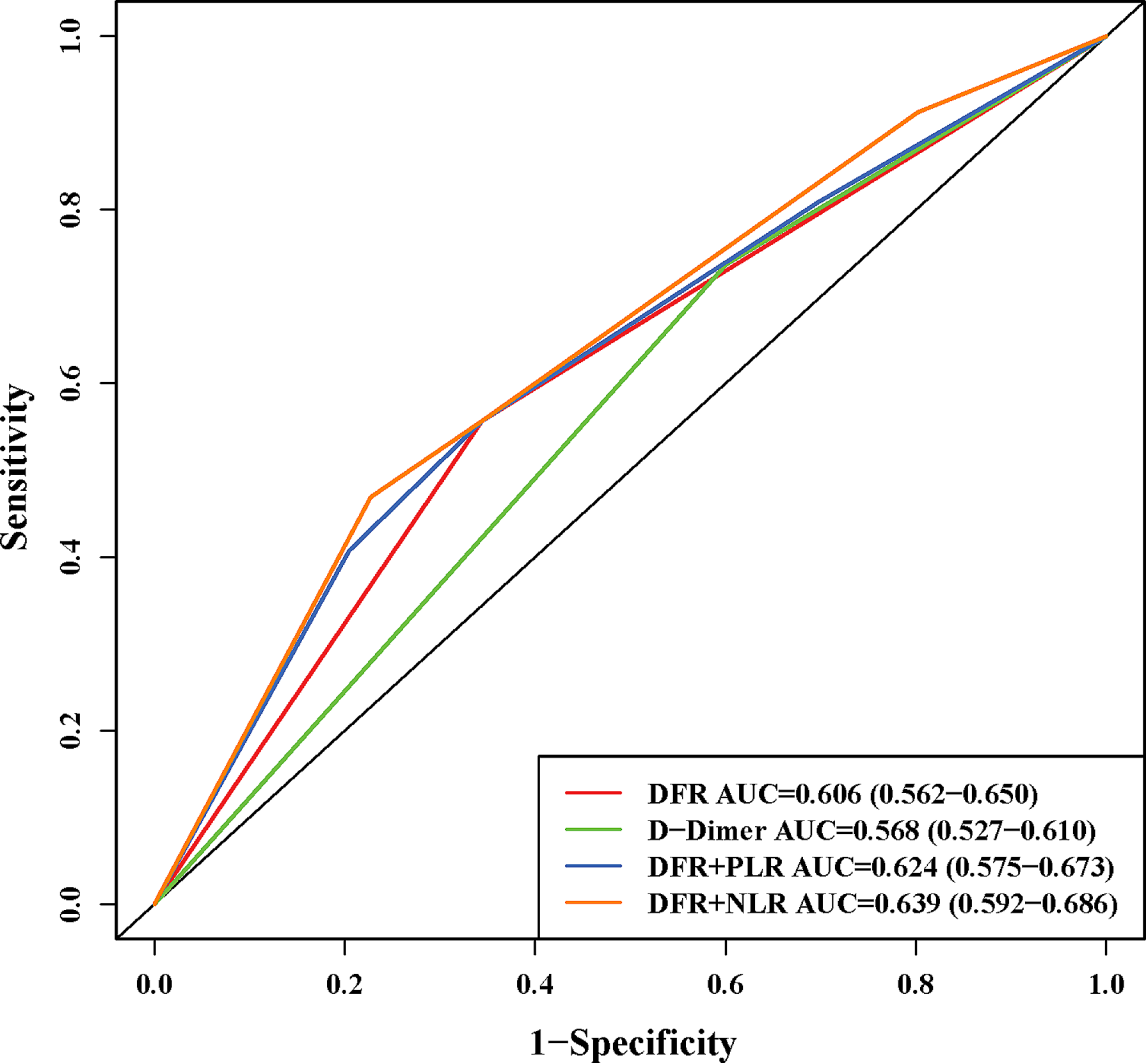
Flow chart of the postpartum women recruited

Between 1 January 2019 and 31 October 2022, 537 postpartum women with suspected PE were enrolled for our study. Screening excluded 21 who did not have D-dimer and fibrinogen measures and 14 who treatment transferred to another hospital. Finally, 502 postpartum women with suspected PE were included for further analysis. Figure 1 shows the flow of women recruited. And Table 1 shows characteristics of postpartum women with suspected PE. Totally, 194 (38.65%) with PE confirmed by CTPA. The mean age was 32.72 (± 4.18) years. Statistical differences were observed between PE and non-PE groups in concomitant with deep vein thrombosis, prothrombin time, DFR, D-dimer, pregnancy weight, systolic blood pressure, diastolic blood pressure, number of fetuses, anticoagulant therapy before PE diagnosed, start time of anticoagulant therapy, and duration of anticoagulant therapy (all *P* < 0.05).

**Table 1:** Characteristics of postpartum women with suspected PE

Variables	Total (*N* = 502)	PE		*P*
		Yes (*n* = 194)	No (*n* = 308)	
Age, years, Mean (± SD)	32.72(± 4.18)	32.93(± 4.13)	32.58(± 4.21)	0.366^a^
Height, cm, Mean (± SD)	161.20(± 4.77)	160.96(± 4.80)	161.36(± 4.76)	0.368^a^
Weight, kg, Mean (± SD)	60.43(± 10.34)	59.74(± 10.81)	60.86(± 10.03)	0.234^a^
BMI, kg/m^2^, Mean (± SD)	23.22(± 3.59)	23.01(± 3.72)	23.35(± 3.51)	0.301^a^
Parturition, n (%)				0.150^d^
Unipara	376(74.9%)	138(71.13%)	238(77.27%)	
Multipara	126(25.1%)	56(28.87%)	70(22.73%)	
Abortion, n (%)				0.409^d^
No	303(60.36%)	122(62.89%)	181(58.77%)	
Yes	199(39.64%)	72(37.11%)	127(41.23%)	
Concomitant with DVT, n(%)				< 0.001^d^
No	446(88.84%)	142(73.2%)	304(98.7%)	
Yes	56(11.16%)	52(26.8%)	4(1.3%)	
Concomitant with hypertension, n (%)				0.263^d^
No	400(79.68%)	160(82.47%)	240(77.92%)	
Yes	102(20.32%)	34(17.53%)	68(22.08%)	
Concomitant with hyperlipidemia, n (%)				1.000^e^
No	496(98.8%)	192(98.97%)	304(98.7%)	
Yes	6(1.2%)	2(1.03%)	4(1.3%)	
Concomitant with diabetes, n (%)				0.678^d^
No	424(84.46%)	166(85.57%)	258(83.77%)	
Yes	78(15.54%)	28(14.43%)	50(16.23%)	
Hb, g/L, M (Q₁, Q₃)	111.00(101.00-120.00)	110.50(102.00-120.00)	112.00(101.00-121.00)	0.970^c^
RBC, 10^12^/L, M (Q₁, Q₃)	3.71(3.42–4.01)	3.68(3.40-4.00)	3.75(3.43–4.01)	0.465^c^
WBC, 10^9^/L, M (Q₁, Q₃)	11.64(9.74–14.34)	11.50(9.76–14.12)	11.80(9.73–14.48)	0.419^c^
PLT, 10^9^/L, Mean (± SD)	174.74(± 63.45)	172.65(± 69.15)	176.05(± 59.67)	0.559^a^
Neutrophil, %, M (Q₁, Q₃)	9.48(7.43–11.72)	9.19(7.82–11.34)	9.62(7.26–12.04)	0.439^c^
LYM, 10^9^/L, M (Q₁, Q₃)	1.35(1.07–1.71)	1.40(1.14–1.73)	1.31(1.04–1.70)	0.117^c^
MONO, 10^9^/L, M (Q₁, Q₃)	0.70(0.49–0.87)	0.69(0.50–0.86)	0.70(0.48–0.88)	0.975^c^
EOS, 10^9^/L, M (Q₁, Q₃)	0.02(0.01–0.05)	0.02(0.01–0.05)	0.02(0.01–0.05)	0.416^c^
BAS, 10^9^/L, M (Q₁, Q₃)	0.02(0.01–0.03)	0.02(0.01–0.03)	0.02(0.01–0.03)	0.213^c^
MPV, fL, M (Q₁, Q₃)	11.00(10.30–11.90)	11.00(10.40–11.90)	11.00(10.30–11.90)	0.807^c^
RDW-CV, %, M (Q₁, Q₃)	13.80(13.10–14.70)	13.70(13.10–14.50)	14.00(13.10-15.03)	0.054^c^
Neutrophil percent, %, M (Q₁, Q₃)	81.50(77.62–84.70)	81.05(77.25–83.90)	81.90(77.88–85.62)	0.085^c^
PT, s, M(Q₁, Q₃)	10.80(10.20-11.38)	10.60(10.00-11.10)	10.90(10.40–11.50)	< 0.001^c^
APTT, s, Mean (± SD)	27.96(± 3.94)	27.65(± 4.00)	28.15(± 3.90)	0.162^a^
TT, s, M (Q₁, Q₃)	15.80(15.10–16.60)	15.70(15.10-16.58)	15.80(15.10-16.62)	0.444^c^
NLR, M (Q₁, Q₃)	6.82(5.01–9.41)	6.62(4.92–8.69)	7.02(5.10–9.95)	0.094^c^
PLR, M (Q₁, Q₃)	124.75(92.40-164.82)	120.25(86.59-157.99)	129.73(97.04-170.88)	0.069^c^
FIB, g/L, M (Q₁, Q₃)	4.11(3.47–4.72)	4.04(3.34–4.62)	4.19(3.55–4.75)	0.161^c^
DFR, mg/g, Mean (± SD)	2.11(± 2.23)	2.53(± 2.43)	1.84(± 2.05)	0.001^b^
D-dimer, mg/L, Mean (± SD)	7.63(± 6.66)	8.97(± 6.91)	6.78(± 6.37)	< 0.001^b^
Pregnancy weight, kg, Mean (± SD)	72.64(± 11.11)	70.79(± 10.95)	73.80(± 11.07)	0.003^a^
SBP, mmHg, Mean (± SD)	124.73(± 14.66)	123.02(± 14.27)	125.80(± 14.81)	0.039^a^
DBP, mmHg, Mean (± SD)	78.30(± 9.93)	77.22(± 9.17)	78.98(± 10.34)	0.047^b^
Gestation, weeks, Mean (± SD)	37.43(± 3.13)	37.57(± 3.29)	37.35(± 3.02)	0.444^a^
Premature delivery, n (%)				0.059^d^
No	374(74.5%)	154(79.38%)	220(71.43%)	
Yes	128(25.5%)	40(20.62%)	88(28.57%)	
Delivery mode, n (%)				0.190^d^
Vaginal delivery	64(12.75%)	30(15.46%)	34(11.04%)	
Cesarean section	438(87.25%)	164(84.54%)	274(88.96%)	
Number of fetuses, n (%)				0.007^d^
1	436(86.85%)	179(92.27%)	257(83.44%)	
2	66(13.15%)	15(7.73%)	51(16.56%)	
Anticoagulant therapy before PE diagnosed, n (%)				< 0.001^e^
No	41(8.17%)	0(0%)	41(13.31%)*^#^	
Pregnancy	9(1.79%)	3(1.55%)	6(1.95%)*	
Postpartum	452(90.04%)	191(98.45%)	261(84.74%)^#^	
Start time of anticoagulant therapy, hours, M (Q₁, Q₃)	24.00(24.00–48.00)	24.00(24.00–72.00)	24.00(21.75-25.00)	< 0.001^c^
Duration of anticoagulant therapy, days, M (Q₁, Q₃)	12.00(8.25-14.00)	15.00(14.00-19.75)	10.00(5.00–12.00)	< 0.001^c^

SD: standard deviation; M: median; Q₁: 1st Quartile; Q₃: 3st Quartile;

^a^ Student’s t test; ^b^ Satterthwaite t test; ^c^ Wilcoxon rank sum test; ^d^ Chi-square test; ^e^ Fisher’s exact test;

PE: pulmonary embolism; BMI: body mass index; DVT: deep vein thrombosis; DFR: D-dimer to fibrinogen ratio; Hb: hemoglobin; RBC: red blood cell; WBC: white blood cell; PLT: platelet; LYM: lymphocyte; MONO: monocyte; EOS: eosinophile; BAS: basophilie; MPV: mean platelet volume; RDW-CV: red blood cell distribution width-coefficient of variation; PT: prothrombin time; APTT: activated partial thromboplastin time; TT: thrombin time; NLR: neutrophil to lymphocyte ratio; PLR: platelet to lymphocyte ratio; FIB: fibrinogen; SBP: systolic blood pressure; and DBP: diastolic blood pressure

### The predictive values of the DFR in postpartum women

**Fig. 2:**
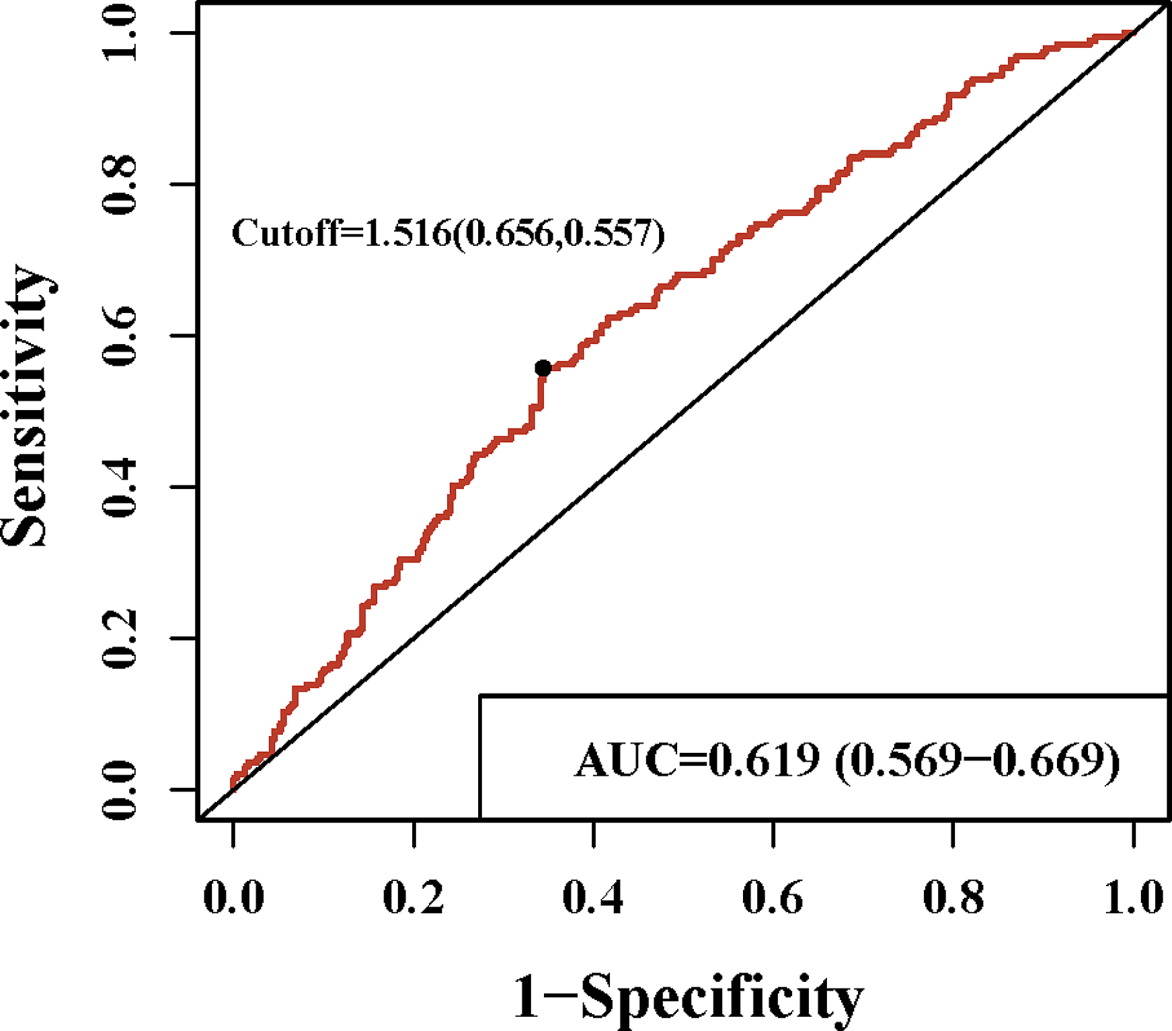
Receiver operating characteristic curves of DFR for the postpartum women

As shown in Fig. 2, ROC curve shows that the AUC of DFR was 0.619 (0.569–0.669). And 1.516 mg/g cut-off level of DFR provided the best discrimination between the PE women and non-PE women. Table 2 shows the relationship between DFR level and odds of PE diagnosis in postpartum women. After adjusting concomitant with deep vein thrombosis, pregnancy weight, premature delivery, start time of anticoagulant therapy, duration of anticoagulant therapy, higher DFR level was associated with lower odds of PE diagnosis (OR: 2.157, 95%CI: 1.290–3.606).

**Table 2:** Association between DFR level and PE patients in postpartum women

Variables	Model1		Model2	
	OR (95%CI)	*P*	OR (95%CI)	*P*
DFR				
Low	Ref		Ref	
High	2.393 (1.656–3.458)	< 0.001	2.157 (1.290–3.606)	0.003

OR: odds ratio; CI: confidence intervals; Ref: reference

Model 1: Crude model

Model 2: Adjusting concomitant with DVT, pregnancy weight, premature delivery, start time of anticoagulant therapy, and duration of anticoagulant therapy

### The predictive values of the DFR combined with NLR or PLR

**Fig. 3:**
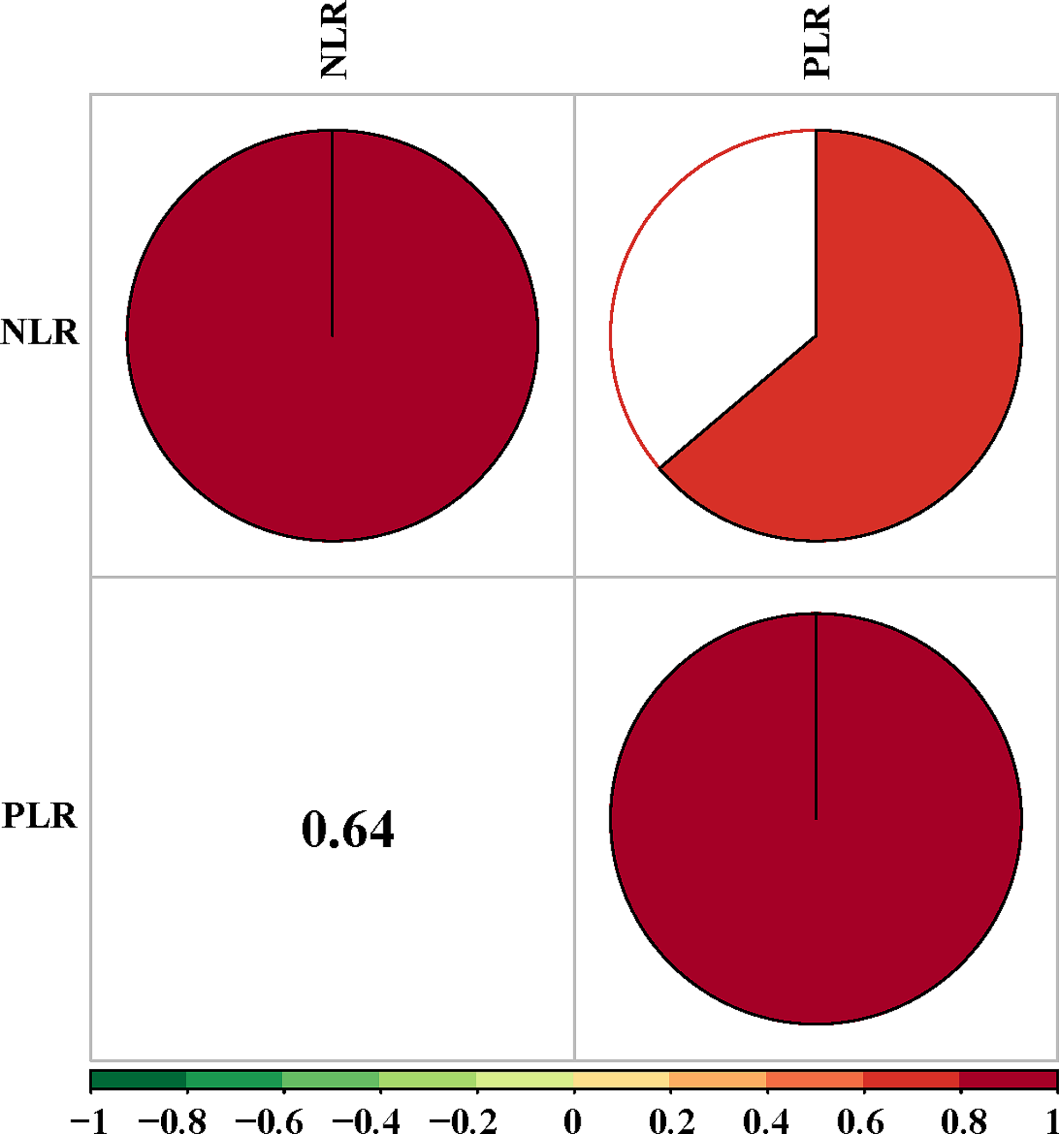
The relationship between PLR and NLR in the postpartum women

**Fig. 4:**
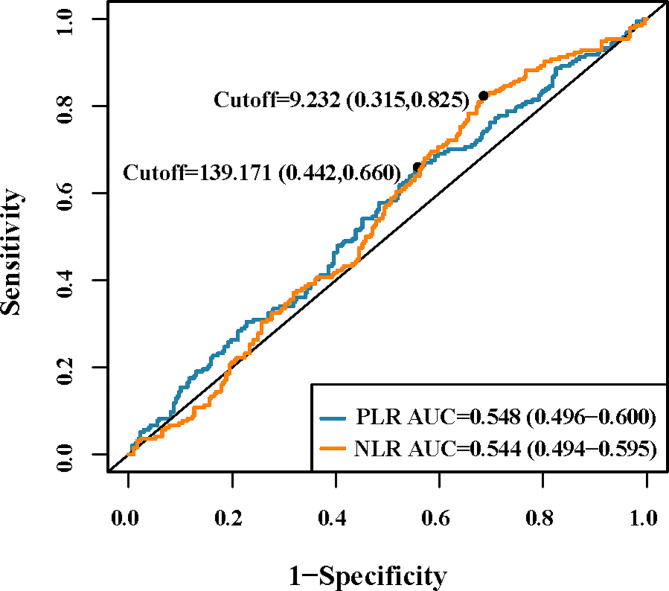
Receiver operating characteristic curves of PLR and NLR

**Fig. 5:**
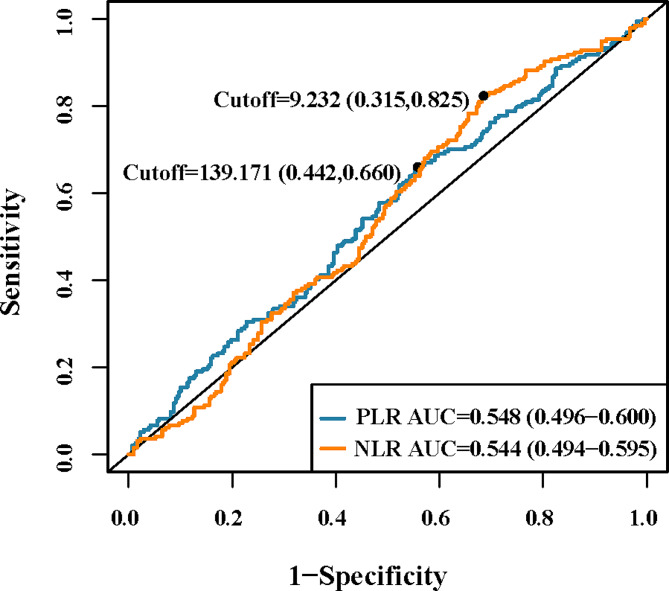
Receiver operating characteristic curves for each biomarker in the postpartum women

Figure 3 shows that PLR was positively correlated with NLR (*r* = 0.64, *P* < 0.05). Then, ROC curve shows that the AUC of PLR and NLR were 0.548 (0.496-0.600) and 0.544 (0.494–0.595) (Fig. 4). The cut-off value of PLR and NLR were 139.171 and 9.232, respectively. The AUC, accuracy, specificity, sensitivity, positive and negative predicted values for each biomarker were shown in Table 3. The ROC curve was established and the AUC of D-dimer, DFR, DFR combined with PLR or NLR were 0.568, 0.606, 0.624, and 0.639, respectively, and the sensitivity and specificity were 0.737, 0.656, 0.557, 0.469, 0.399, 0.557, 0.656, and 0.773 respectively. The result indicates that PLR and NLR can increase the diagnostic value of DFR. The ROC curves for each biomarker were shown in Fig. 5.

**Table 3:** The diagnostic value of each biomarker in postpartum women

Index	D-Dimer	DFR	DFR and PLR	DFR and NLR
AUC (95% CI)	0.568 (0.527–0.610)	0.606 (0.562–0.650)	0.624(0.575–0.673)	0.639(0.592–0.686)
Accuracy (95% CI)	0.530 (0.485–0.574)	0.618 (0.573–0.660)	0.618(0.573–0.660)	0.655(0.612–0.697)
Specificity (95% CI)	0.399 (0.345–0.454)	0.557 (0.487–0.627)	0.656(0.603–0.709)	0.773(0.726–0.820)
Sensitivity (95% CI)	0.737 (0.675–0.799)	0.656 (0.603–0.709)	0.557(0.487–0.627)	0.469(0.399–0.539)
PPV (95% CI)	0.436 (0.382–0.490)	0.701 (0.649–0.754)	0.505(0.438–0.572)	0.565(0.489–0.642)
NPV (95% CI)	0.707 (0.639–0.775)	0.505 (0.438–0.572)	0.701(0.649–0.754)	0.698(0.649–0.747)

DFR: D-dimer to fibrinogen ratio; PLR: platelet to lymphocyte ratio; NLR: neutrophil to lymphocyte ratio; AUC: area under the curve; CI: confidence interval; PPV: positive predictive value; NPV: negative predictive value

## Discussion

Our findings suggest that the DFR may be a valuable biomarker for diagnosing PE in postpartum women. We identified a DFR cut-off value of 1.516 mg/g, showing superior diagnostic performance compared to D-dimer alone. Furthermore, combing DFR with PLR or NLR enhanced diagnostic accuracy beyond that achieved by DFR alone.

Our finding indicated that the diagnostic value of DFR exceeded that of D-dimer alone in diagnosing PE in postpartum women, consistent with prior research highlighting DFR’s potential as a valuable diagnostic tool for thromboembolic events [20, 21]. This enhanced accuracy may be attributed to the complementary roles of D-dimer in fibrinolysis and fibrinogen in clot formation, integrated within DFR to provide a comprehensive assessment of coagulation abnormalities associated with PE [15, 22, 23]. Compared to DFR, D-dimer levels exhibit reduced diagnostic precision due to physiological increase during normal pregnancy [11]. In a study on progressive hemorrhagic injury, the DFR ratio emerges as a potential predictor of venous thrombosis [24]. The predictive value of DFR has been reported in lower extremity deep vein thrombosis (LEDVT) among young patients with cerebral hemorrhage [21]. The superior diagnostic value of DFR suggests its utility as a valuable instrument for enhancing the prompt and precise diagnosis of PE in postpartum women. Our findings imply that DFR could function as a reliable adjunctive tool in the diagnostic assessment of postpartum PE, potentially minimizing unnecessary imaging and optimizing timely intervention. Further validation through larger prospective studies is essential to establish DFR’s utility across diverse clinical settings and populations.

Furthermore, our study indicates that combining DFR with either the PLR or NLR significantly enhances diagnostic accuracy for PE in postpartum women. Incorporating inflammatory markers like NLR and PLR alongside DFR likely improves the discriminatory capability of the diagnostic model by capturing the interplay between coagulation and inflammation process in PE pathophysiology. Both PLR and NLR serve as systemic inflammation and are associated with increased thrombotic risk [25, 26]. Previous research has shown that combining PLR and DFR with the Wells score yields high specificity in predicting LEDVT in young patients with cerebral hemorrhage patients [21]. Integrating inflammatory markers into the diagnostic algorithm alongside DFR offers a more comprehensive assessment of the multifactorial nature of PE in postpartum women.

Our findings carry significant clinical implications for the diagnostic approach to PE in postpartum women. Specifically, the DFR, identified with a cut-off value of 1.516 mg/g, suggests a superior diagnostic value compared to D-dimer alone in this population. Integrating DFR into routine diagnostic protocols could potentially enhance the timely and accurate identification of PE, thereby facilitating prompt intervention and reducing unnecessary imaging procedures. Moreover, our study highlights the complementary role of DFR when combined with PLR or NLR. This synergistic approach may further improve diagnostic precision, offering clinicians a more comprehensive tool for evaluating suspected PE cases in postpartum patients. Future research should focus on validating these findings in larger prospective studies across diverse clinical settings to establish the reliability and applicability of DFR as a diagnostic biomarker for PE in postpartum women.

For the first time, we provide a novel DFR-based clinical calculator for predicting the probability of PE diagnosis in postpartum women. We identified an optimal cut-off of 1.516 mg/g where DFR demonstrates utility in PE prediction. Our study has limitations, including its cross-sectional design which precludes causal inference and longitudinal predictive assessment of DFR in postpartum PE. Moreover, the relatively small sample size may limit generalizability. Further multicenter prospective studies with larger cohorts are warranted to validate the diagnostic utility of DFR.

## Conclusion

For postpartum women, the DFR emerges as a valuable biomarker for diagnosing PE, potentially reducing unnecessary testing. DFR is of greater value in excluding PE when combined with NLR or PLR. Implementing DFR may help identify high-risk postpartum women, guiding clinicians in treatment decisions and potentially improving outcomes.

## Data Availability

The datasets used and/or analyzed during the current study are available from the corresponding author on reasonable request.

## Abbreviations

PE: Pulmonary embolism
DFR: D-dimer to fibrinogen ratio
NLR: Neutrophil-to-lymphocyte ratio
PLR: Platelet-to-lymphocyte ratio
BMI: Body mass index
AUC: Area under curve
ORs: Odds ratios
Cis: Confidence intervals
ROC: Receiver-operator characteristic
